# Olfactory impairment in patients with primary Sjogren’s syndrome and its correlation with organ involvement and immunological abnormalities

**DOI:** 10.1186/s13075-021-02624-6

**Published:** 2021-09-29

**Authors:** Xue Xu, Linyu Geng, Chen Chen, Wentao Kong, Baojie Wen, Wei Kong, Siwen Chen, Huayong Zhang, Jun Liang, Lingyun Sun

**Affiliations:** 1 Department of Rheumatology and Immunology, Nanjing Drum Tower Hospital, The Affiliated Hospital of Nanjing University Medical School, No. 321 Zhongshan Road, Nanjing, 210008 Jiangsu China; 2 Department of Clinical Nutrition, Nanjing Drum Tower Hospital, The Affiliated Hospital of Nanjing University Medical School, No. 321 Zhongshan Road, Nanjing, 210008 Jiangsu China; 3 Department of Ultrasound, Nanjing Drum Tower Hospital, The Affiliated Hospital of Nanjing University Medical School, No. 321 Zhongshan Road, Nanjing, 210008 Jiangsu China

**Keywords:** Primary Sjogren’s syndrome, Olfaction, Impairment, Inflammation, Cognition

## Abstract

**Objective:**

Patients with autoimmune diseases often present with olfactory impairment. The aim of the study was to assess the olfactory functions of patients with primary Sjögren’s syndrome and to correlate these findings with their disease activity.

**Methods:**

Fifty-two patients with primary SS and 52 sex- and age-matched healthy control subjects were included. All of them underwent clinical and laboratory examination. Olfactory functions were evaluated using olfactory function assessment by computerized testing including the three stages of smell: threshold, identification, and memory of the different odors.

**Results:**

All the olfactory scores (olfactory threshold, identification, and memory) in patients with pSS were significantly decreased than the control group (all *P* < 0.01). Patients had higher proportion of anosmia (13.5% vs 0%) and hyposmia (19.2% vs 11.5%) than controls (*χ*^2^ = 10.526, *P* < 0.01). Multivariable regression analysis revealed that ESSDAI and the symptoms of dryness, fatigue, and limb pain had negative influence on olfactory function (adjusted *R*^2^ = 0.381, 0.387, 0.513, and 0.614, respectively). ESSPRI showed significantly negative association with olfactory threshold, identification, memory, and total scores. Olfactory identification and memory scores were decreased in pSS patients with thyroid dysfunction or hypocomplementemia (*P* < 0.05). Smell threshold scores were decreased in pSS patients with anti-SSA antibody or anti-nuclear antibody compared with those without those autoantibodies (*P* < 0.01).

**Conclusion:**

Our findings indicate that olfactory functions are impaired in pSS patients. There was a close correlation between olfactory dysfunction and disease severity and immunological abnormalities. Immune and systemic inflammation dysregulation might play a role in the mechanism of this defect.

**Supplementary Information:**

The online version contains supplementary material available at 10.1186/s13075-021-02624-6.

## Background

Sjögren’s syndrome (SS) is a chronic autoimmune disorder marked by lymphocytic infiltration of exocrine glands, mainly salivary and lacrimal, causing xerostomia and keratoconjunctivitis sicca [1]. Other submucous glands distributed in the stomach, nose, and vagina can also be involved and lead to decreased exocrine function even interfere with individual’s quality of life. In addition, a variety of extraglandular disease manifestations can occur, including fatigue, fever, myalgia, arthritis, and mild cognitive dysfunction [2]. The prevalence of the disease is 1–3%; however, most patients live without the diagnosis due to a slow progression of the inflammation. The disease can be divided into primary (pSS) and secondary (sSS) Sjögren’s syndrome.

Recent evidences suggest that autoimmune diseases predispose to an absence of smell function and diminished smell sensitivity. This has been demonstrated in the patients with systemic lupus erythematosus [3], rheumatoid arthritis, and systemic sclerosis [4, 5]. However, there is some controversial information about the olfactory function of pSS patients. Previous studies indeed report impairment in patients with SS of smell. The earliest evidence of an impaired sense of smell in patients with SS was available since 1972 when Heinkin et al. found that 29 patients with xerostomia and rhinitis sicca due to SS or other causes had significant decreased smell acuity [6]. Kamel et al. studied 28 SS patients and 37 controls and observed a reduction in smell threshold in the SS group compared with controls [7]. A significantly lower olfactory identification scores in pSS patients than controls was reported [8, 9]. It might be anticipated that progressive exocrine gland damage with loss of secretions would affect the special senses of smell, whereas other results arise with different conclusions. One group reported that smell discrimination test was abnormal in 2 (2.6%) and 1 (1.3%) in pSS patients and controls, but the difference was statistically insignificant [10]. Another group interviewed 36 patients with pSS and demonstrated no differences in smell threshold between patients and controls [11]. Some of these studies used the same method to assess olfactory function in patients but have yielded contradictory results. At present, there is no study that conducts a complete examination of olfactory threshold and identification in pSS patients. Therefore, further complete and precise investigations about olfaction in pSS patients are needed.

The aim of this study was to determine the olfactory function including smell threshold, identification, and memory in a larger cohort of patients with pSS by computerized testing. We also wanted to investigate a potential relationship between disease activity and olfactory capacity, the impact of different organ manifestations, and immunological parameter on the olfactory functions and evaluate whether examination of the sense of smell has diagnostic or predictive value in primary SS.

## Methods

### Patients and controls

Fifty-two Chinese patients with pSS (49 women and 3 men) from the Department of Rheumatology and Immunology at Nanjing Drum Tower Hospital were enrolled in the present study. All the patients fulfilled the American-European Consensus group criteria for pSS [12]. Fifty-two age- and gender-matched healthy Chinese individuals were included from the community as normal controls. Both controls and patients with pSS were required to be between 20 and 70 years of age. This study was approved by the Ethics Committee of the Nanjing Drum Tower Hospital and was carried out in compliance with the Helsinki Declaration. All participants provided informed consent before enrollment. Subjects with previous head injury, head and neck surgery, active nasal-sinus infections, allergic diseases, stroke, radiotherapy to head and neck, chemotherapy, nasal polyposis, and anosmia were excluded from the study, since these diseases could affect an appropriate interpretation of results.

### Clinical assessment

Complete medical histories, physical examinations, and laboratory tests were collected. All the data were collected in a standardized computerized electronically filled form including demographics, past medical history with date of diagnosis, past surgeries, comorbidities, smoking habits, and current therapies. Organ involvement was defined according to the criteria described previously: pulmonary fibrosis = bibasilar fibrosis on chest radiography and high-resolution CT, pulmonary arterial hypertension (PAH) = pulmonary arterial pressure (PAP) ≥ 35 mmHg (transthoracic echocardiography), thrombocytopenia = platelet count below the lower limit of normal value, abnormal thyroid function = abnormal thyroid hormone levels, hyperglobulinemia = the levels of serum globulin IgG or IgM above the upper limit of normal value, and hypocomplementemia = the levels of serum complement C3 or C4 below the lower limit of normal value. All the patients completed the European League Against Rheumatism (EULAR) Sjögren’s Syndrome Patient Reported Index (ESSPRI) (mean score of 0–10 numerical scales for pain, fatigue, and dryness features, including oral, ocular, and global dryness) [13]. Physicians completed the EULAR Sjögren’s Syndrome Disease Activity Index (ESSDAI) according to the criteria as described previous [14].

### Laboratory tests

Routine hematologic and biochemical tests were performed, and the results were analyzed in the hospital’s laboratory. Plasma was tested for antinuclear antibodies (ANA) using indirect immunofluorescence with HEp-2 cells as the antigen substrate (Immuno Concepts, Sacramento, USA). The presence of anti-SSA and anti-SSB antibodies was confirmed by enzyme-linked immunosorbent assay (ELISA) (Quanta Lite ENA 6; Inova Diagnostics). Rheumatoid factor (RF) was determined using an enzyme-linked immunoassay (Euroimmun, Medizinische Labor diagnostic, AG).

### Subjective assessment of smell

Self-reported perception of sense of smell was obtained prior to olfactory testing. Participants were asked to score their own general subjective smell perception on a visual analogue scale (VAS), and the score ranged from 0 to 10 (where 0 meant complete loss of smell perception while 10 meant excellent perception of smell). Scores below 5 were considered dysosmia (distorted smell perception).

### Olfactory assessment

All the participants were blind to the examiner using Olfactory Function Assessment by Computerized Testing (OLFACT-C^TM^, Osmic Enterprises, Inc. www.osmicenterprises.com). The pipe from which the odor was delivered was positioned approximately 2 cm in front of the participant’s nostrils for approximately 3 s. It comprises tests for odor threshold (THR), odor identification (ID), and odor memory (ME). The olfactory threshold test (score range, 1–13.5) was determined based on a series of binary dilutions of the n-butanol solution in light mineral oil. Scores of 8–10 were considered normosmia (normal olfactory sensitivity), and scores of 4–7 were identified as hyposmia (reduced ability to smell), while scores of 1–3 signified anosmia (complete loss of smell), and scores above 10 indicated sensitive olfaction. The olfactory identification and memory tests respectively consisted of two tasks and assessed one’s ability to identify and remember odors. In task A (score range, 0–10), the participant was presented with 10 odors in sequence and was asked to identify each one from 4 choices. Then, the test broke for 10 min before starting task B (score range, 0–20); the participant was presented with 20 odors, including the 10 old odors from part A and 10 new odors. The participant was asked to identify each odor from 4 choices and also indicate whether it was old or new odor (old odors and new odors score range, 0–10, respectively). Together, the three tests took approximately 60 min. At the end of the testing procedure, the sum of the THR, ID, and ME scores was referred to as the TIM (threshold + identification + memory).

This score ranged from 1 to 63.5 points.

### Statistical analysis

The data collected were coded, edited, and the statistical analysis was performed using SPSS software (version 20.0, SPSS Inc., Chicago, IL, USA). Data were presented as mean ± standard deviation. An independent samples *t* test was used for comparing normally distributed continuous variables in the patient and control groups. A chi-square test was used to compare dichotomous variables, and Pearson’s correlations were used to measure the strength and direction of linear relationships between pairs of continuous variables. To assess factors independently associated with olfactory function, four multivariate linear regression analyses were performed for olfactory threshold (THR), identification (ID), memory (ME), and olfactory total score (TIM). On the basis of previous knowledge and confounding assessment, we selected the following covariates in the multivariate linear regression models: age (continuous), sex (male = 1, female = 2), education (continuous), disease duration (continuous), ESSPRI (continuous), ESSDAI (continuous), and ESR (continuous). All differences were considered significant at *P* < 0.05.

## Results

### Patient cohort

Patient’s characteristics are summarized in Table 1; 52 patients with pSS were investigated. There were 3 (5.77%) males (ages ranged from 36 to 49 years, mean age of 42.33 ± 6.51 years) and 49 (94.23%) females (ages ranged from 21 to 70 years, mean age of 47.67 ± 12.81 years). The duration of the disease ranged from 0.1 to 20 years, with mean duration of 4.53 ± 4.64 years. For the control group, there were 4 males (ages ranged from 32 to 51 years, mean age of 41.03 ± 8.18 years) and 48 females (ages ranged from 20 to 68 years, mean age of 48.69 ± 11.75 years). No controls were taking medication known to interfere with smell. There was no history of smoking and alcohol consumption in any of both groups. The two groups did not differ significantly in their social status and education. Concerning the systemic inflammation parameters, pSS patients had a mean ESR value of 34.38 ± 21.39 mm/h and a mean CRP value of 11.95 ± 10.36 mg/L. Thirty patients (57.7%) had increased levels for ESR (cut off value < 20 mm/h) and 16 patients (30.8%) had elevated values for the CRP (cut off value < 8.0 mg/L).

**Table 1:** Clinical characteristics of primary Sjogren’s syndrome patients

	Patients	Controls
	(*n* = 52) (100%)	(*n* = 52) (100%)
Age (years)	47.36 ± 12.45	49.09 ± 11.48
Sex (female)	49 (94.23%)	48 (92.31%)
Duration of disease (years)	4.53 ± 4.64	NA
Education (years)	9.32 ± 4.91	11.05 ± 5.37
Smoking	0	0
Objective ocular involvement	45 (86.54%)	
Objective oral involvement	40 (76.92%)	
Positive salivary gland biopsy	39 (75%)	
ESR (mm after 1 h)	34.38 ± 21.39	
CRP (mg/L)	11.95 ± 10.36	
ANA	43 (82.69%)	
Anti-SSA antibody	41 (78.84%)	
Anti-SSB antibody	15 (28.85%)	
RF	13 (25%)	
Current treatment		
Corticosteroids	40 (76.92%)	
Hydroxychloroquine	32 (61.54%)	
Cyclophosphamide	10 (19.23%)	
Cyclosporine	12 (23.07%)	
Methotrexate	2 (3.85%)	
Mycophenolate mofetil	4 (7.69%)	
*Tripterygium wilfordii*	6 (11.54%)	
Other immunosuppressant agent	2 (3.85%)	
Disease activity indexes		
ESSPRI	3.14 ± 1.63	
ESSDRI	9.17 ± 6.08	

Results are expressed as mean ± SD or number (%). *ESR*, erythrocyte sedimentation rate, *CRP* C-reactive protein, *ANA* antinuclear antibody, *RF* rheumatoid factor, *ESSDAI* EULAR SS disease activity index, *ESSPRI* EULAR SS patient reported index

### Smell test results of SS patients

A significant decrease in olfactory function was observed in patients affected with pSS compared to healthy controls (Table 2). In fact, patients had a significantly lower smell threshold score compared with healthy controls (*P* = 0.001). Lower scores were also observed among patients with pSS compared with controls for both olfactory identification and olfactory memory (*P =* 0.001). Meanwhile, overall olfactory sum (TIM scores) were significantly reduced in pSS cohort compared to the healthy control group, as illustrated in Table 2.

**Table 2:** Olfactory behavior test

	pSS patients	Controls	*P* value
	*n* = 52	*n* = 52	
Olfactory threshold	8.89 ± 3.79	10.97 ± 2.42	0.001
Odor identification test	19.13 ± 5.27	23.84 ± 2.45	0.001
Task A (10 odors)	6.76 ± 1.93	8.41 ± 1.12	0.001
Task B (20 odors)	12.36 ± 3.71	15.44 ± 1.87	0.001
Odor memory test	12.88 ± 3.54	15.67 ± 2.20	0.001
Old 10 odors	6.05 ± 2.26	7.73 ± 1.45	0.001
New 10 odors	6.38 ± 2.32	7.94 ± 1.52	0.001
Total TIM scores	40.91 ± 9.82	50.49 ± 5.35	0.001

Results are expressed as mean ± SD. *TIM* threshold + identification + memory

**Fig. 1:**
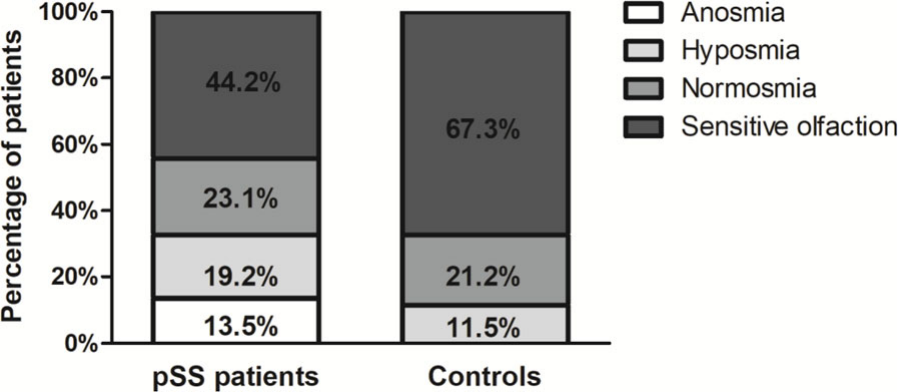
Higher rates of anosmia and hyposmia in pSS patients than controls. Olfactory function of pSS patients and controls, stratified into anosmia (olfactory threshold scores of 1–3), hyposmia (olfactory threshold scores of 4–7), normosmia (olfactory threshold scores of 8–10), and sensitive olfaction (olfactory threshold scores > 10) individuals. A chi-square test was used (*χ*^2^ = 10.526, *P* < 0.01)

Furthermore, anosmia was present exclusively in the pSS group (13.5% of patients vs 0% of controls), and hyposmia was more prevalent in patients with pSS than in controls (19.2% of patients vs 11.5% of controls). Normal sense of smell seemed to be similar between the two groups; however, sensitive olfaction was more prevalent in controls than in patients with pSS (67.3% of controls vs 44.2% of patients) (*χ*^2^ = 10.526, *P* < 0.01) (Fig. 1). Our results suggest that pSS patients had higher rates of anosmia and hyposmia and lower rates of sensitive olfaction.

### Subjective assessment of smell of pSS patients

Participants rated their subjective olfactory function on a visual analogue scale from 0 to 10. The pSS group had significantly lower mean subjective olfactory scores than the control group (6.09 ± 2.83 vs 8.25 ± 1.94, *P* < 0.01). In the pSS group, self-evaluations of olfactory scores correlated significantly with measured olfactory scores (*r* = − 0.682, *P* = 0.003) (Supplementary Fig. 1a). Twenty-nine (55.76%) pSS patients were dysosmia (self-reported olfactory scores < 5), and the proportion was much lower than 17 (32.69%) patients (olfactory threshold scores < 8) confirmed by objective examination (*χ*^2^ = 6.613, *P =* 0.018) (Supplementary Fig. 1b).

### Correlation of the smell test results of pSS patients with their disease activity

**Fig. 2:**
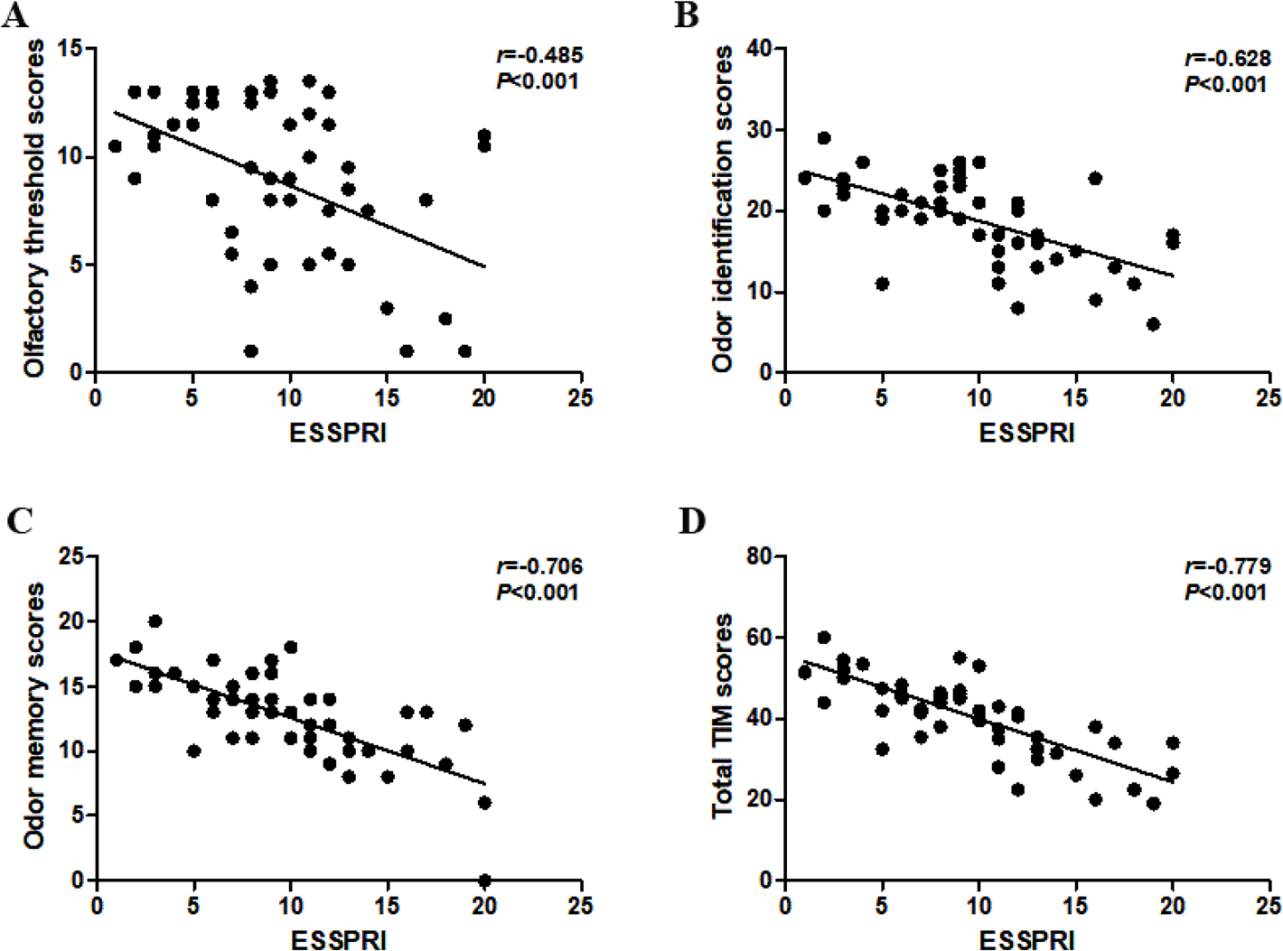
Negative associations of olfactory function scores with ESSPRI in pSS patients. **a** ESSPRI was correlated with olfactory threshold scores in pSS patients. **b** ESSPRI was correlated with olfactory identification scores in pSS patients. **c** ESSPRI was correlated with olfactory memory scores in pSS patients. **d** ESSPRI was correlated with olfactory TIM scores in pSS patients

By correlating the olfactory function with disease activity, as assessed by ESSPRI, ESSDAI, and ESR, the associations between the scores of olfactory tests and the clinical and laboratory variables are shown in Table 3. Multivariable regression revealed that ESSDAI were associated with worse performance in olfactory threshold test (*β* = − 0.266, *P* = 0.046). ESSPRI also showed a negative effect on olfactory threshold, identification, memory, and TIM scores. Olfactory function did not correlate with duration of disease, nor with education or ESR. The presence of dryness in patients had a negative influence on olfactory threshold, memory, and TIM scores, symptoms of fatigue also showed a negative effect on olfactory threshold, identification, and TIM scores, and the presence of limb pain was associated with lower olfactory identification and memory scores in multivariable models (Supplementary table 1). Moreover, ESSPRI showed significantly negative association with olfactory threshold, identification, memory, and TIM scores (*r* = − 0.485 and *P <* 0.001, *r* = − 0.628 and *P <* 0.001, *r* = − 0.706 and *P <* 0.001, *r* = − 0.779 and *P <* 0.001, respectively) (Fig. 2a–d). Our results suggested that the loss of olfaction was associated with disease activity.

**Table 3:** Associations between olfactory tests and selected clinical and laboratory variables in pSS patients

	THR	ID(A + B)	ME(o + n)	TIM
Age				
*β*	0.059	− 0.068	− 0.104	0.073
*P*	0.722	0.656	0.454	0.671
Sex				
*β*	− 0.147	0.061	− 0.085	− 0.055
*P*	0.241	0.591	0.413	0.541
Education				
*β*	0.185	0.168	0.044	0.178
*P*	0.269	0.27	0.749	0.141
Disease duration				
*β*	0.022	0.114	0.061	0.092
*P*	0.864	0.337	0.576	0.327
ESSPRI				
*β*	− 0.447	− 0.587	− 0.652	− 0.724
*P*	0.002	0.001	0.001	0.001
ESSDAI				
*β*	− 0.266	− 0.102	0.191	− 0.089
*P*	0.046	0.434	0.112	0.386
ESR				
*β*	0.207	0.041	− 0.096	0.068
*P*	0.127	0.738	0.393	0.484
Adjusted *R*^2^	0.261	0.384	0.486	0.618

Multivariable regression analysis was used to evaluate relations between different parameters. *THR* threshold, *ID* identification, *ME* memory, *TIM* threshold + identification + memory, *ESSPRI* EULAR SS Patient Reported Index, *ESSDAI* EULAR SS disease activity index, *ESR* erythrocyte sedimentation rate

### Smell test results of the patients in association with their different clinical and immunological manifestations

**Fig. 3:**
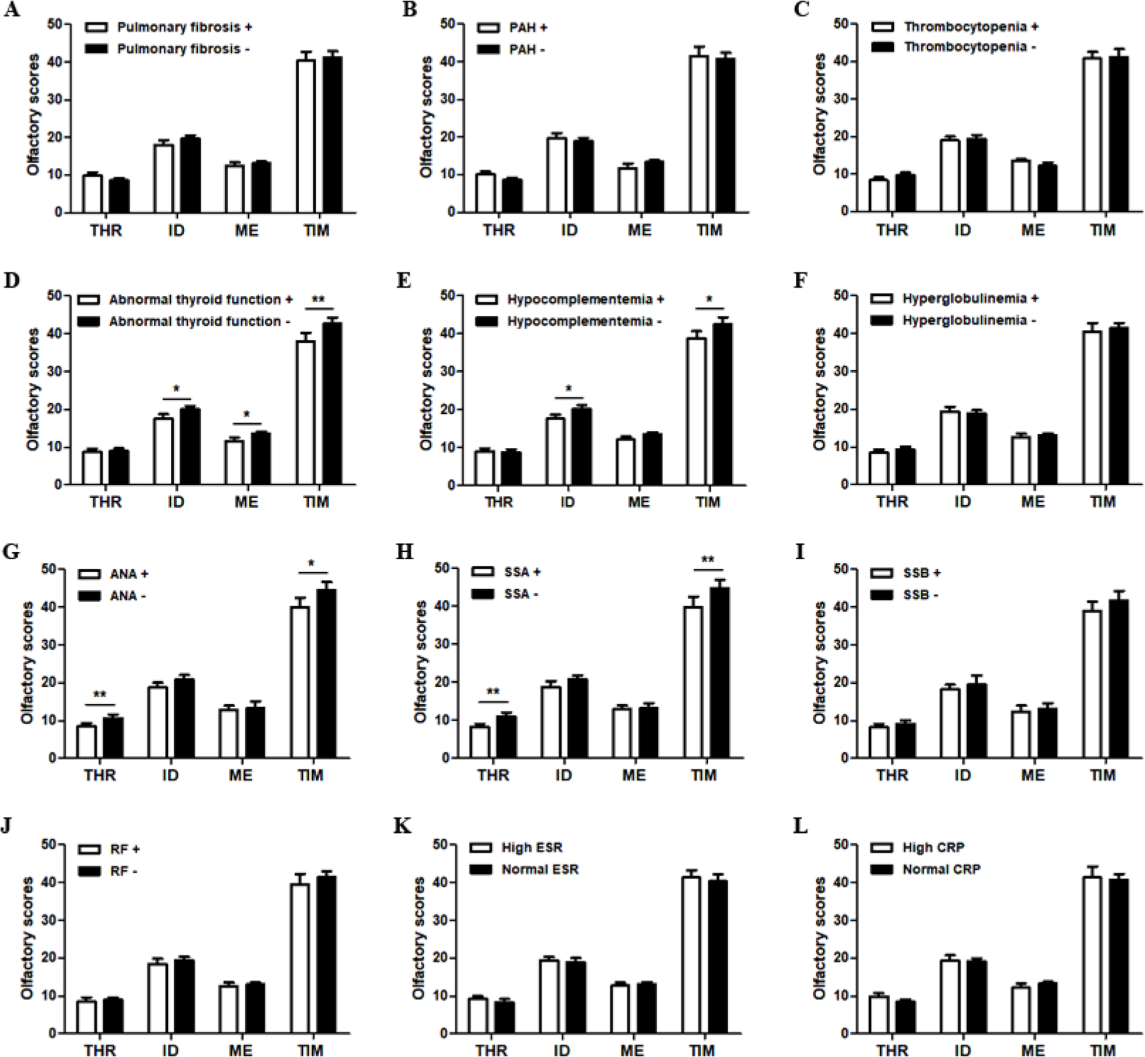
Association between smell test results of the pSS patients and clinical manifestations and immunological parameter. **a–f** Olfactory THR, ID, ME, and total TIM scores in the presence and absence of pulmonary fibrosis (**a**), pulmonary arterial hypertension (**b**), thrombocytopenia (**c**), abnormal thyroid function (**d**), hypocomplementemia (**e**), and hyperglobulinemia (**f**) in pSS patients. **g–j** Olfactory THR, ID, ME, and total TIM scores in the presence and absence of ANA (**g**), anti-SSA antibody (**h**), anti-SSB antibody (**i**), and RF (**j**) in pSS patients. **k** Olfactory THR, ID, ME, and total TIM scores in pSS patients with high ESR or normal ESR levels. **l** Olfactory THR, ID, ME, and total TIM scores in pSS patients with high CRP or normal CRP levels. THR, threshold; ID, identification; ME, memory; TIM, threshold + identification + memory. Results represent the means ± SEM. *P < 0.05, **P < 0.01

Consequently, the correlation between olfactory functions and clinical and laboratory parameter was assessed in pSS patients. Patients with an involvement of the thyroid showed significantly diminished scores for olfactory ID, ME and total TIM scores (*P* < 0.05, *P* < 0.05, and *P* < 0.01, respective) (Fig. 3d). Olfactory ID and total TIM scores were decreased in pSS patients with hypocomplementemia compared with those without (*P* < 0.05). Olfactory THR and TIM scores were significantly lower in patients with antinuclear antibody (*P* = 0.006; *P* = 0.027) and anti-SSA antibody (*P* = 0.002; *P* = 0.009) than in patients without those autoantibodies (Fig. 3g, h). There was no correlation of smell test scores between pSS patients with anti-SSB antibody and RF and those without (Fig. 3i, j).

### Influence of treatment on olfactory function

Comparing the smell test results of the pSS patients treated with different therapies, we found patients under therapy with glucocorticoid showed a tendency for reduced THR scores compared with the patients not taking glucocorticoid (*P* = 0.063) (Supplementary Fig. 2a). Comparing different dosages of oral steroids did not reveal significant differences for smell scores (data not show). Treatment with hydroxychloroquine did not correlate with olfactory function (Supplementary Fig. 2b). No significant difference was observed in treatment with immunosuppressants associated with changes in olfactory scores (Supplementary Fig. 2c).

## Discussion

In this study, we present the first investigation that combined a detailed testing of the olfactory threshold, identification, and memory with a comprehensive rheumatological assessment of disease activity in a cohort of 52 pSS patients. Four major results were obtained: (1) pSS patients exhibit significantly deteriorated olfactory function, with 13.5% of subjects suffering from anosmia and 19.2% suffering from hyposmia. (2) ESSDAI and ESSPRI had negative impact on olfactory function and impaired smell function were associated with several organ involvement and autoantibodies. (3) The proportion of dysosmia in patients evaluated by subjective assessment was higher than determined by objective examination. (4) Olfactory function was not significantly affected by pSS treatment. Together, these data collectively highlight the reduction of olfactory function in pSS patients correlate with disease activity and could have useful value in diagnosis of primary SS.

Peripheral olfactory function is to some degree represented by threshold scores and involvement of the olfactory receptor cells, whereas smell identification and memory reflect higher central nervous processing of odors, which requires complex skills including cognitive and verbal process [15]. Therefore, for full assessment of olfaction, all 3 stages of the smell test are important. Each stage is directed at a different ability and impairment and may help identify specific problems among diseases. In the literature, previous studies have only analyzed olfactory threshold or olfactory identification in pSS patients using Sniffin’ Sticks, University of Pennsylvania Smell Identification test, or other methods. We choose to use the OLFACT-C™ computerized test due to its ability in identifying different aspects of smell dysfunction and regulating the duration and concentration of odor steadily, and thus, this instrument can provide reliable and valid data [16]. Our findings are in line with previous research in which the impairment of smell function in patients with pSS has been reported [5–8, 17] and contradictory to two studies [9, 10]. A possible reason behind the contradiction may be related to the difference in methods of assessing smell function and sample size of patients. In the current study, changes in three subtests might indicate that not only peripheral olfactory system but also central nervous olfactory organ were impaired. The olfactory function deficits can be attributed directly or indirectly to many reasons. Lymphocytic infiltration of nasal glands can lead to atrophy, decreased secretion, and nasal dryness, which may be the major reason for the olfactory impairment. But nasal airway inflammation, which can cause edema, thickening of nasal mucosa, and damage of nerve fibers, may affect contact between odor molecules and olfactory receptor cells, rendering them less sensitive to stimuli. Unfortunately, few studies concerning the effects of nasal mucosal dryness on impaired olfactory function have been reported. Recently, a study showed that pSS patients exhibited nasal cavity dryness; however, this did not affect olfactory function [18].

ESSDAI and ESSPRI are two disease activity indexes developed by the European League Against Rheumatism (EULAR) SS task force. Concerning symptoms, we found an inverse association between olfactory function and ESSPRI scores. Saliva and nasal mucus are important for maintaining the normal function of taste buds and patients with reduced salivary secretion are known to have olfactory function abnormalities [9]. Dryness accounts for a large proportion of the ESSPRI scoring system. ESSDAI measures 12 organ-specific domains and was widely used to assess disease activity of pSS. In the present study, negative correlation between olfactory function and ESSPRI were found. This result suggested that dysosmia may be one of the manifestations of active pSS. Both innate and adaptive immune systems are upregulated in pSS patients, resulting in the production of several pro-inflammatory cytokines and autoantibodies [19, 20]. Increased levels of pro-inflammatory cytokines such as interleukin 6 [21], interferon-γ [22], tumor necrosis factor-α [23], and interleukin 1β [24] reduce hippocampi and amygdalae neurogenesis and impair the proliferation of olfactory neurons. Several other studies have shown an association between inflammation and decreased olfactory impairment [25]. Therefore, the reduced central nervous olfactory function might be explained by the active systemic inflammation and neuroinflammation in the central nervous system (CNS). More studies at the molecular level, investigating the inflammatory activity in patients with pSS and its effect on the peripheral or central smell organs, are necessary to understand the occurrence of the disorder in patients with pSS.

Furthermore, olfactory function scores were significantly reduced in patients with thyroid involvement or hypocomplementemia, compared to those without organ involvement. Hypocomplementemia and hypothyroidism are important characteristic of the disease. Olfactory impairment is known to be influenced by thyroid function, as adult hypothyroid humans and mice can lose their sense of smell [26, 27]. FT3 levels have been found to have a more important association with olfactory parameters than TSH or FT4 levels. Study showed that adult mice become hypothyroid with propylthiouracil with olfactory impairment [28]. This effect was prevented by daily administration of T4. Because T4 is essential for the normal maturation of the nervous system, it may also be essential for the proliferation of new olfactory receptor neurons. In a prospective randomized interventional study enrolled with 32 primary hypothyroid patients and 31 controls, significant improvement in smell and taste functions were demonstrated in comparison of scores at the third month of treatment and before treatment of hypothyroid patients [29]. Thus, the authors suggest that the future workup of patients with smell/taste loss should include investigations for thyroid functions. Besides, some of the researchers hypothesized that olfaction disorders reduce olfactory stimulation and diminished olfactory stimulus may trigger hypothyroidism [30]. Here, we also demonstrated impairment of olfactory function in pSS patients with hypothyroidism, which confirmed the association of hypothyroidism with olfactory impairment. Complement activation may play a role in the formation of C5b-9 terminal complex promoting demyelination that could lead to smell impairment [31]. Previously, patients with hereditary angioedema (HAE), similar to SLE, which is characterized by the activation of the classical complement pathway with C4 consumption, had demonstrated impaired sense of smell associated with C4 levels [32]. Here, in our study, olfactory impairment was also found in our pSS patients with hypocomplementemia, supporting the role of complement activation in promoting smell impairment in pSS patients, but the exact mechanism requires further investigation in the near future.

In addition, impaired smell function was also found to be correlated with various immunological parameters in pSS patients, including the presence of ANA and anti-SSA antibody. It is reported that some antibodies are capable of binding and penetrating neuronal cells of the limbic areas (hippocampus and amygdala) that are associated with olfaction [33]. Reduction of the sense of smell could be induced in lupus animal models by passive transfer of anti-ribosomal P antibodies into the CNS [34, 35]. In addition, antibodies directed against N-methyl-D-aspartate receptor subtype NR2 (anti-NR2) could cause neuronal death manifested as reduced hippocampal gray matter in pSS patients [36]. Thus, antibodies can interfere with central olfactory organ, which suggested autoimmune mediated mechanisms may play a role in the pathogenesis of olfactory dysfunction.

The pSS group had significantly lower self-reported olfactory scores and showed strong associations with objective smell function, which was consistent with pioneer reports [37]. This indicated that the olfactory disorder is somatic in nature and consistent with the patients’ own experiences. Moreover, the proportion of dysosmia in patients evaluated by subjective assessment (55.76%) was higher than determined by objective examination (32.69%). Since loss of smell in pSS patients and particularly its impact on quality of life has tended to be overlooked in the past, our results suggest that olfactory function testing should be part of the routine assessment of patients with pSS and physicians should be aware of these abnormalities and should inform their patients about these manifestations and about possibilities to increase olfactory sensations to increase quality of life.

Most of the medications used in our cohort, including corticosteroid, hydroxychloroquine, cyclophosphamide, and methotrexate, have been reported to be associated with impaired olfactory functions [38]. We found there were no significant differences regarding the smell THD, ID, ME, or TIM scores when comparing the different medications used in patients. However, there was a tendency for THR scores to be slightly lower in pSS patients taking corticosteroid, which possibly indicated that the patients who needed additional steroids therapy had a higher disease activity and therefore had limitations in their olfactory function caused by the ongoing systemic autoimmune response.

To our best knowledge, this is the first study to utilize olfactory memory test to evaluate the ability of pSS patients to remember odors that were previously presented. In the smell assessment task B, after each odor presentation, the participant was asked to identify the odor (semantic memory) and then indicate whether it was old or new (episodic memory). Our results showed that both semantic memory and episodic memory were impaired in pSS patients (Table 2), which suggested that these patients had potential cognitive dysfunction. Extraglandular manifestations are commonly reported in pSS, including peripheral or central nervous system involvement [39]. CNS manifestations associated with pSS include focal central lesions, encephalitis, movement disorders, and problems with memory, cognition, and depression [40]. In pSS, the incidence rate of cognitive disorders is not uniform and varies across literature from 11 to 100% [41]. Mechanism of cognition and memory dysfunction in pSS is explained by immune-mediated inflammatory small-vessel disease or direct infiltration of the brain tissue with chronic inflammatory cells [42]. In some instances, olfactory impairment can precede cognitive dysfunction by several years and have been associated with anxiety, depression, and other neuropsychiatric manifestations [43, 44]. Despite the large subclinical of pSS-associated cognitive dysfunction, there are no guidelines for screening for cognitive disorder nor is there consensus regarding criteria for the diagnosis of cognitive impairment in patients. Therefore, the olfactory test could be used as an indicator to predict whether cognitive impairment occurs.

The current study provides evidence of olfactory impairment in pSS patients. However, there are some limitations of the study. First, due to time and cost constraints, intranasal Schirmer test was not used to evaluate nasal cavity dryness. Another limitation is the small sample size. However, we believe the current study provides preliminary support for designing a larger, more focused effort. Finally, the patients and controls did not undergo repeat olfactory behavior tests to determine whether the olfactory deficits have improved before and after effective treatment. Further investigation should be performed using larger samples, longitudinal assessment, cognitive evaluations, and a combination of odor-induced functional MRI measurements and smell test for this population.

## Conclusions

In conclusion, our results demonstrated a significant reduction in the olfactory abilities of pSS patients, which correlated with disease activity. Although the exact mechanism of olfactory impairment has yet to be elucidated, the possibility of an immune/inflammation-mediated mechanism is intriguing. Therefore, physicians are encouraged to perform routine smell test in patients with pSS and educate them on methods to cope with it for better quality of life.

## Supplementary Information

**Additional file 1: Supplementary Fig. 1** Lower subjective olfactory scores in pSS patients than controls. **Supplementary Fig. 2** Impact of different treatment on olfactory function. **Supplementary Table 1** Associations between olfactory tests and selected clinical and laboratory variables in pSS patients

## Data Availability

The datasets used and/or analyzed during the current study are available from the corresponding author on reasonable request.

## Abbreviations

PSS: Primary Sjögren’s syndrome
ESSPRI: European League Against Rheumatism (EULAR) Sjögren’s Syndrome Patient Reported Index
ESSDAI: EULAR Sjögren’s Syndrome Disease Activity Index
THR: Threshold
ID: Identification
ME: Memory
TIM: Threshold + identification + memory
