# Supplementary material for: Olfactory impairment in patients with primary Sjogren’s syndrome and its correlation with organ involvement and immunological abnormalities
Source: Arthritis Res Ther. 2021 Sep 29;23:250. doi: 10.1186/s13075-021-02624-6 (PMC8480095; doi:10.1186/s13075-021-02624-6)
Supplement: Supplementary file 1 — Additional file 1: Supplementary Fig. 1 Lower subjective olfactory scores in pSS patients than controls. Supplementary Fig. 2 Impact of different treatment on olfactory function. Supplementary Table 1 Associations between olfactory tests and selected clinical and laboratory variables in pSS patients [file 13075_2021_2624_MOESM1_ESM.doc]

**Supplementary Fig. 1** Lower subjective olfactory scores in pSS patients than controls


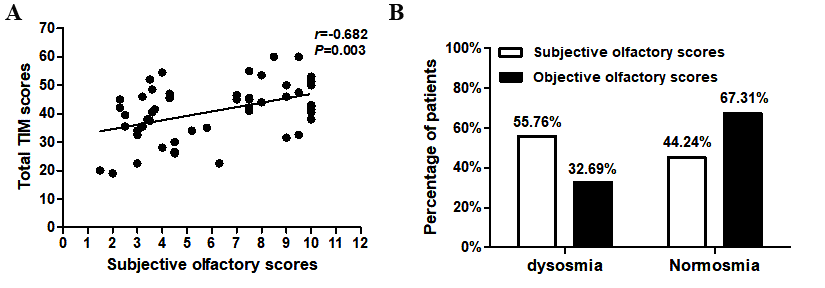


**Fig. 1** Lower subjective olfactory scores in pSS patients than controls. **a** Correlation between self-reported olfactory scores and objective olfactory scores in patients with pSS. Statistical significance was analyzed by the Pearson’s correlation test. **b** The percentage of dysosmia and normosmia in pSS patients determined by subjective and objective olfactory assessment. TIM: threshold + identification + memory.

**Supplementary Fig. 2** Impact of different treatment on olfactory function


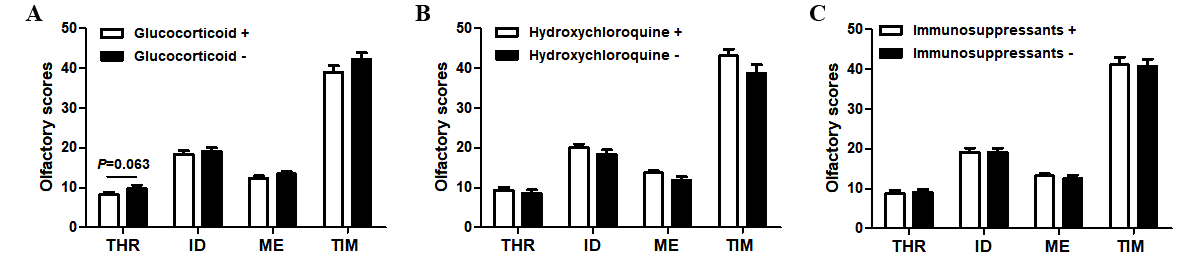


**Fig. 2** Impact of different treatment on olfactory function. **a** Olfactory THR, ID, ME and total TIM scores in pSS patients with taking glucocorticoid or not. **b** Olfactory THR, ID, ME and total TIM scores in pSS patients with taking hydroxychloroquine or not. **c** Olfactory THR, ID, ME and total TIM scores in pSS patients with taking immunosuppressants or not. THR：threshold；ID identification；ME：memory；TIM: threshold + identification + memory. Results represent are the Mean ± SEM.

**Supplementary table 1** Associations between olfactory tests and selected clinical and laboratory variables in pSS patients

|  | THR | ID(A+B) | ME(o+n) | TIM |
| --- | --- | --- | --- | --- |
| Age |  |  |  |  |
| *β* | 0.192 | -0.031 | -0.197 | -0.014 |
| *P* | 0.195 | 0.827 | 0.131 | 0.904 |
| Sex |  |  |  |  |
| *β* | -0.142 | 0.087 | -0.093 | -0.042 |
| *P* | 0.243 | 0.458 | 0.383 | 0.652 |
| Education |  |  |  |  |
| *β* | 0.236 | 0.23 | -0.044 | 0.199 |
| *P* | 0.126 | 0.125 | 0.742 | 0.095 |
| Disease duration |  |  |  |  |
| *β* | 0.058 | 0.056 | 0.11 | 0.092 |
| *P* | 0.669 | 0.669 | 0.352 | 0.375 |
| Dryness |  |  |  |  |
| *β* | -0.454 | -0.117 | -0.319 | -0.353 |
| *P* | 0.005 | 0.423 | 0.021 | 0.004 |
| Fatigue |  |  |  |  |
| *β* | -0.371 | -0.357 | -0.226 | -0.417 |
| *P* | 0.006 | 0.006 | 0.052 | 0.001 |
| Limb pain |  |  |  |  |
| *β* | 0.216 | -0.273 | -0.332 | -0.183 |
| *P* | 0.115 | 0.042 | 0.007 | 0.084 |
| CRP |  |  |  |  |
| *β* | 0.143 | -0.003 | -0.174 | 0.009 |
| *P* | 0.228 | 0.981 | 0.098 | 0.921 |
| Adjusted R^2^ | 0.34 | 0.381 | 0.492 | 0.61 |

Multivariable regression analysis was used to evaluate relations between different parameters. THR: threshold; ID: identification; ME: memory; TIM: threshold + identification + memor; CRP: C reactive protein.
